# Cardioprotective role of diacerein in diabetic cardiomyopathy via modulation of inflammasome/caspase1/interleukin1β pathway in juvenile rats

**DOI:** 10.1007/s00210-023-02921-8

**Published:** 2024-01-15

**Authors:** Marwa M. M. Refaie, Hanaa Hassanein Mohammed, Elshymaa A. Abdel-Hakeem, Asmaa M.A. Bayoumi, Zamzam Hassan Mohamed, Sayed Shehata

**Affiliations:** 1 Department of Medical Pharmacology, Faculty of Medicine, Minia University, El-Minia, 61511 Egypt; 2 Department of Histology and Cell Biology, Faculty of Medicine, Minia University, El-Minia, 61511 Egypt; 3 Department of Medical Physiology, Faculty of Medicine, Minia University, El-Minia, 61511 Egypt; 4 Department of Biochemistry, Faculty of Pharmacy, Minia University, El-Minia, 61511 Egypt; 5 Department of Pediatric, Faculty of Medicine, Minia University, El-Minia, 61511 Egypt; 6 Department of Cardiology, Faculty of Medicine, Minia University, El-Minia, 61511 Egypt

**Keywords:** Diacerein, Diabetes, Cardiomyopathy, Interleukin 1 beta

## Abstract

Diabetes mellitus is a common metabolic disorder affecting different body organs; one of its serious complications is diabetic cardiomyopathy (DCM). Thus, finding more cardiopreserving agents to protect the heart against such illness is a critical task. For the first time, we planned to study the suspected role of diacerein (DIA) in ameliorating DCM in juvenile rats and explore different mechanisms mediating its effect including inflammasome/caspase1/interleukin1β pathway. Four-week-aged juvenile rats were randomly divided into groups; the control group, diacerein group, diabetic group, and diabetic-treated group. Streptozotocin (45 mg/kg) single intraperitoneal (i.p.) dose was administered for induction of type 1 diabetes on the 1st day which was confirmed by detecting blood glucose level. DIA was given in a dose of 50 mg/kg/day for 6 weeks to diabetic and non-diabetic rats, then we evaluated different inflammatory, apoptotic, and oxidative stress parameters. Induction of DCM succeeded as there were significant increases in cardiac enzymes, heart weights, fasting blood glucose level (FBG), and glycosylated hemoglobin (HbA1c) associated with elevated blood pressure (BP), histopathological changes, and increased caspase 3 immunoexpression. Furthermore, there was an increase of malondialdehyde (MDA), inflammasome, caspase1, angiotensin II, nuclear factor kappa-B (NF-κB), tumor necrosis factor-α (TNFα), and interleukin 1β (IL1β). However, antioxidant parameters such as reduced glutathione (GSH) and total antioxidant capacity (TAC) significantly declined. Fortunately, DIA reversed the diabetic cardiomyopathy changes mostly due to the observed anti-inflammatory, antioxidant, and anti-apoptotic properties with regulation of blood glucose level.DIA has an ability to regulate DCM-associated biochemical and histopathological disturbances.

**Supplementary Information:**

The online version contains supplementary material available at 10.1007/s00210-023-02921-8.

## Introduction

Diabetes mellitus is a common metabolic disorder characterized by hyperglycemia, insulin deficiency, and/or insulin resistance along with disturbance in lipid metabolism. Diabetic cardiomyopathy (DCM) is considered as one of the life-threatening complications that face diabetic patients at all ages. Evidence documented an association between earlier age of type 1 diabetes and premature onset cardiovascular comorbidities (Schäfer et al., 2020). DCM is accompanied with disturbances of myocardial cell structure and function, left ventricular hypertrophy, myocardial cell fibrosis, and death (Liu et al., 2023). Failure of the heart muscle is the most frequent feature causing mortality of those patients (Zhang et al., 2022, Akhtar et al., 2023).

Till now, the cellular and molecular mechanisms underlying DCM are not fully understood. There are multifactorial complex pathophysiological processes involving oxidative stress, neurohormone activation, impaired homeostasis, mitochondrial damage, inflammation, and lipid peroxidation of cell membrane with disturbed normal cell functions (Yan et al., 2020, Zhang et al., 2022). In addition, there are different signaling molecules including nuclear factor kappa-B (NF-κB), tumor necrosis factor α (TNFα), and interleukin 1β (IL1β) that stimulate excessive production of pro-inflammatory cytokines causing cardiac inflammation and induce more formation of free radicals, DNA damage with replication errors, and improper DNA repair (Zhu et al., 2019, Yao et al., 2021).

Cardiac inflammation is a remarkable reaction in the setting of DCM that is mainly related to NLR family pyrin domain Containing 3 (NLRP3) inflammasome. Nod-like receptors (NLRs) are a type of pattern recognition receptor family that recognizes damage/danger-associated molecular patterns and pathogen-associated molecular patterns (Luo et al., 2017, Ding et al., 2019). NLRs are classified into four different subfamilies, but the most involved one in elaborating inflammasome complex is NLRP subfamily, specifically NLRP3 (Ding et al., 2019, Dhalla et al., 2020). Increased blood glucose level is a strong stimulus of inflammasome/caspase1/interleukin1β pathway that is vigorously involved in the pathogenesis of DCM; thus, it is assumed that controlling this signaling cascade could prevent the progression of the disease (Luo et al., 2017, Ding et al., 2019, Dhalla et al., 2020).

Diacerein (DIA) is one of the naturally occurring anthraquinone derivatives that has non-steroidal anti-inflammatory properties and used mainly in treatment of osteoarthritis (Martorell et al., 2021). The principal pharmacological and physiological role of DIA is its ability to inhibit IL1β system and the downstream signaling pathways. In addition, DIA has other biological activities as it could suppress oxidative stress, inflammatory, apoptotic, and catabolic processes, but it has pro-anabolic properties. (Almezgagi et al., 2020, Fouad et al., 2020). Different previous studies revealed the cardioprotective effect of DIA in controlling myocardial infarction, attenuating left ventricular remodeling, angiotensin II (Ang II)-induced cardiomyopathy, and chronic stress-induced cardiac dysfunction. In these models, DIA succeeded to ameliorate the associated inflammation, vascular dysfunction, disturbed renin angiotensin aldosterone system, and hazards of sympathetic nervous system overactivity. Moreover, DIA could stimulate endothelial nitric oxide synthase and diminish different pro-inflammatory cytokines leading to a reduction of high blood pressure (Agarwal et al., 2021, He et al., 2021, Jangsiripornpakorn et al., 2022, Silva et al., 2022, Wang et al., 2022b).

Based on the above data, it seems that there is an important relation between increasing the release of IL1β and occurrence of heart damage in diabetic patients. Meanwhile, DIA has the ability to modulate different pathways involved in mediating cardiac injury in those patients. These factors directed our attention to evaluate the suspected preserving effect of DIA in a model of diabetic cardiomyopathy and study the different mechanisms mediating it.

## Materials and methods

### Ethics

Faculty of Pharmacy of Minia University, Egypt, approved the current research in accordance with ARRIVE guidelines and EU Directive 2010/63/EU guidelines for animals. The approval number is MPEC (230801).

### *The study protocol*

We purchased 40 male young weaned rats of Wistar albino species aged 4 weeks, and their body weights ranged from 90 to 100 g. The animals were from the Animal Research Centre, Giza, Egypt, and the acclimatization period was for 1 week before starting the experiment. Suitable stainless steel cages were used for keeping rats (3 rats/cage); animals were freely supplied with chow and tap water; the humidity was 40%; the temperature was 24 ± 2 °C with exposure to 12-h dark/light cycle.

DIA was emulsified daily in a suspension of 1% carboxymethylcellulose immediately before administration.

Animals were randomly divided into four groups (*n* =10 in each group).

Group Ӏ (CON): The control group that was given the vehicle orally (carboxymethylcellulose) for a period of 6 weeks.

Group II (DIA): The DIA-administered group that was given DIA in a dose of 50 mg/kg/day (Refaie et al., 2022) orally for a period of 6 weeks.

Group III (diabetes): The diabetic group which was administered streptozotocin (STZ) (45 mg/kg) single i.p. dose on the 1st day (An et al., 2022).

Group IV (DIA+ diabetes): The diabetic-treated group given STZ (45 mg/kg) single i.p. dose on the 1st day (An et al., 2022) plus DIA in a dose of 50 mg/kg/day (Refaie et al., 2022) orally for a period of 6 weeks.

### *Chemicals*

DIA was purchased from EVA Pharma Co., Egypt, and STZ was from Sigma Aldrich Co., USA. Total antioxidant capacity (TAC) kit was from Biodiagnostic Co., Egypt, (Catalog # TA2513). ELISA kits of inflammasome (Catalog # MBS2033695); Ang II (Catalog # MBS705139); caspase 1 (Catalog # MBS2510133); TNFα (Catalog # MBS2507393); NF-κB (Catalog # MBS453975); cardiac enzymes: lactate dehydrogenase (LDH), troponin I, creatine kinase-MB (CK-MB), and glycosylated hemoglobin (HbA1c) kit, with catalog numbers (Catalog # MBS043166), (Catalog # MBS722833), (Catalog # MBS2515061), (Catalog # MBS2033689), respectively, were from My BioSource Co., San Diego, CA, USA. IL1β mouse monoclonal antibody was from Santa Cruz Biotechnology, Inc., Germany, (Catalog # sc-12742). GAPDH rabbit monoclonal antibody was from Cell Signaling Technology, Inc., USA, (Catalog # 2118).

### *Measuring of fasting blood glucose level (FBG) and glycosylated hemoglobin (HbA1c) level*

Following administration of a single i.p. injection of STZ (45 mg/kg) by 72 h, rats were fasted overnight and FBG level in each rat was measured. Only diabetic animals with FBG level ≥ 16.7 mmol/L were included in this study. At the end of our experiment, HbA1c status was evaluated by the available commercial kit.

### *Measurement of blood pressure (BP)*

Just before termination of the study, blood pressure of each rat was measured using the tail-cuff method by LETICA, Panlab S.L., Barcelona, Spain. First, each rat was kept at 38 °C for about 15 min for detecting the pulsation of tail artery followed by applying tail-cuff to measure BP for five successive times (Miguel et al., 2005).

### *Collection and storage of samples*

At last, we anesthetized the rats by injecting a single dose of urethane hydrochloride (1 g/kg) i.p. Blood was obtained from the abdominal aorta, then samples were centrifuged at 1792 g for 15 min (JanetzkiT30 centrifuge, Germany). The heart of each rat was separated, washed adequately, and weighed, then a longitudinal section was performed. Ten percent formalin was used for fixation of tissue then embedded in paraffin for the histopathological examination. The remaining parts were homogenized in a Glas-Col homogenizer at speed of 1008 g for 20 min. Each homogenate sample was separated and stored at −80 °C for the biochemical analysis.

### *Biochemical analysis*

#### *Measuring cardiac enzymes, TNFα, Ang II, NF-κB, inflammasome, and caspase 1*

The cardiac enzymes; CK-MB, LDH, and troponin I along with the inflammatory mediators; TNFα, NF-κB, inflammasome, caspase 1, and Ang II were detected by ELISA kits according to the manufacturers’ instructions.

#### *Evaluating oxidative stress parameters*

Membrane lipid peroxidation was evaluated by detecting thiobarbituric acid–reacting substance and expressed as an equivalent to MDA, using 1,1,3,3-tetramethoxypropane as a standard in a unit of nmol/mg tissue (Buege and Aust, 1978, Mihara and Uchiyama, 1983).

We measured reduced glutathione (GSH) by the available calorimetric method that depends on the binding of the sulfhydryl group with Ellman’s reagent resulting in formation of a yellow color measured by Beckman DU-64 UV/VIS spectrophotometer, USA, at 405 nm in a unit of nmol/mg tissue (Moron et al., 1979). Also, total antioxidant capacity (TAC) was detected calorimetrically in a unit of mmol/L.

#### *Western blotting of IL1β*

Western blotting analysis was performed to evaluate IL1β expression in each cardiac tissue (Ewees et al., 2019). Simply, samples were homogenized and then separated on a 10 % SDS–PAGE gel. Protein bands were blotted to a nitrocellulose membrane by a semi-dry blotter (Bio-Rad). The blot was blocked, probed overnight at 4 °C with IL1β antibody or GAPDH antibody, and incubated with alkaline phosphatase-tagged secondary antibodies for 1 h at room temperature. Each blot was analyzed by 5-bromo-4-chloro-3-indolyphosphate and nitro-blue tetrazolium colorimetric detection method (Sigma-Aldrich Co., USA). These bands were analyzed by Image-J and GraphPad Prism-5 software programs.

### *Histopathological evaluation*

After completion of the experiment, samples were gathered, and the hearts were cut meticulously. The tissues were directly fixed in neutral buffered 10% formalin solution and handled into 5-μm-thick paraffin sections and stained with hematoxylin and eosin (H&E) in addition to Masson’s trichrome stain (Azar et al., 2019). We examined the stained sections of the heart using the light microscope.

### *Staining technique for immunohistochemical studies*

Immunocytochemical staining was performed by polyclonal rabbit antibodies for anti-cleaved caspase 3 (Catalog # PA1- 26426) which were obtained from Sigma Aldrich. Paraffin sections of different groups were sliced into 5-μm thickness and incubated at 42 °C in an oven for 24 h. The sections were deparaffinized in xylol (1 h), hydrated in descending grades of ethyl alcohol, and then incubated in hydrogen peroxide (5 min). We washed the sections two times in PBS (5 min each). The primary antibody (diluted 1:100) was put to the sections, then they were incubated for 1.5 h. Afterwards, the sections were washed two times in PBS for 5 min each. The secondary antibody (diluted 1:1000) was put, and the sections were incubated for 20 min, then washed three times in PBS for 5 min. Diaminobenzidine tetra hydrochloride solution was put to the sections, and they were incubated for 10 min. The sections were washed in distilled water and counterstained with Mayer’s hematoxylin (2 min), following that they were washed in tap water, dehydrated, cleared, and mounted by DPX (Alsharif et al., 2023).

#### Photography

For inspecting and capturing images of the histological and immunohistochemical sections, an Olympus light microscopy (Olympus, Japan) was used. Slides were photo’d by using Olympus digital camera (U.TV0.5XC-3). Photos were saved as jpg and handled by Adobe Photoshop 7.

#### Morphometric study

The analysis of cardiac histopathology was scored depending on the degree of injury detected in each group using semiquantitative measurement: 0 = no lesions; 1 = mild (1–25%); 2 = moderate (26–45%); and 3 = severe (> 45%).

For Masson trichrome sections, five sections of every single heart were scored. The degree of myocardial fibrosis was ranked and scored as follows: 0 = no fibrosis; 1+ = fibrosis involving < 25% of the myocardial interstitial; 2+ = fibrosis involving 25–50%; 3+ = fibrosis involving 50–75%; and 4+ = fibrosis involving 75 to 100%.

#### Measuring area fraction of caspase 3

Image J 22 software (open source Java image processing program) was used for area fraction measurement of the activated caspase 3 immune-positivity. We measured the area fraction in a standard measuring frame per 5 photomicrographs in each group using a magnification ×400 by light microscope conveyed to the monitored screen. Areas containing positively immunostained tissues were used for estimation regardless the intensity of staining (Goyal et al., 2016, Yue-Chun et al., 2016) .

### *Statistical analysis*

One-way ANOVA was used to evaluate our results, then Tukey’s multiple comparison test was performed, and data were expressed as mean ± SD using GraphPad Prism software (version 5). The significance was considered when the calculated *p* value was less than 0.05.

## Results

### Effect of DIA on heart weights and cardiac enzymes

Heart weights and cardiac enzymes significantly increased in the diabetic group compared to both the control and diabetic-treated groups. However, the above mentioned parameters significantly decreased on co-administration of DIA compared to the diabetic-untreated group (Table 1).

**Table 1:** Effect of DIA on heart weights and cardiac functional enzymes

Groups	Heart weights (mg)	Troponin I (ng/mL)	CK-MB (U/L)	LDH (U/L)
CON	306.0 ± 25.2	0.8 ± 0.1	42.0 ± 7.3	150.6 ± 8.8
DIA	335.6 ± 39.8	0.7 ± 0.1	32.6 ± 5.9	164.5 ± 12.0
Diabetes	443.1 ± 35.9^ac^	6.9 ± 1.2^ac^	60.7 ± 9.8^ac^	271.1 ± 18.0^ac^
DIA+diabetes	334.6 ± 51.0^b^	1.3 ± 0.2^b^	44.4 ± 7.2^b^	189.6 ± 19.3^ab^

Results of current study were for 10 observations represented as mean ± SD. Significance was considered if *p* < 0.05

*CON* control group, *DIA* diacerein, *CK-MB* creatine kinase MB, *LDH* lactate dehydrogenase

^a^It was given if significance was found compared to the control group

^b^It was given if significance was found compared to the diabetic group

^c^It was given if significance was found compared to the treated diabetic group

### *Effect of DIA on FBG, HbA1c, Ang II, and BP*

FBG, HbA1c, Ang II, and BP significantly increased in the diabetic group if compared to both the treated diabetic group and the control group. However, DIA administration to diabetic rats could significantly reverse these increased levels in comparison to the diabetic-untreated group (Table 2).

**Table 2:** Effect of DIA on FBG, HbA1c, Ang II, and BP

Groups	FBG (mmol/L)	HbA1c (%)	Ang II (pg/mL)	BP (mm Hg)
CON	7.3 ± 1.2	4.5 ± 0.7	200.3 ± 13.6	107.1 ± 8.0
DIA	6.9 ± 1.0	5.1 ± 0.8	206.9 ± 21.0	111.7 ± 9.9
Diabetes	21.6 ± 3.2^ac^	8.6 ± 1.1^ac^	516.0 ± 61.9^ac^	164.5 ± 12.1^ac^
DIA+diabetes	13.4 ± 2.4^ab^	6.5 ± 1.0^ab^	235.4 ± 30.8^ab^	137.0 ± 15.7^ab^

Results of current study were for 10 observations represented as mean ± SD. Significance was considered if *p* < 0.05

*CON* control group, *DIA* diacerein, *FBG* fasting blood glucose level, *Ang II* angiotensin II, *BP* blood pressure

^a^It was given if significance was found compared to the control group

^b^It was given if significance was found compared to the diabetic group

^c^It was given if significance was found compared to the treated diabetic group

### *Effect of DIA on oxidative stress parameters*

MDA and GSH were measured in heart tissue while TAC was measured in serum. Results showed significant increases of MDA levels, while GSH and TAC significantly diminished in the diabetic group compared to the control group and diabetic-treated group. This effect could be significantly reversed by co-administration of DIA compared to the diabetic-untreated group (Table 3).

**Table 3:** Effect of DIA on oxidative stress parameters

Groups	MDA (nmol/mg tissue)	GSH (nmol/mg tissue)	TAC (mmol/L)
CON	4.1 ± 0.7	7.7 ± 0.6	0.9 ± 0.08
DIA	5.1 ± 0.7	6.9 ± 1.2	0.7 ± 0.12
Diabetes	11.4 ± 2.2^ac^	1.7 ± 0.3^ac^	0.5 ± 0.06^ac^
DIA+diabetes	8.0 ± 1.3^ab^	6.1 ± 0.7^b^	0.7 ± 0.05^ab^

Results of current study were for 10 observations represented as mean ± SD. Significance was considered if *p* < 0.05

*CON* control group, *DIA* diacerein, *MDA* malondialdehyde, *GSH* reduced glutathione, *TAC* total antioxidant capacity

^a^It was given if significance was found compared to the control group

^b^It was given if significance was found compared to the diabetic group

^c^It was given if significance was found compared to the treated diabetic group

### *Effect of DIA on inflammasome, caspase 1, TNFα, and NF-κB*

Measurement of these parameters in heart tissue showed significant increases of inflammasome, caspase 1, TNFα, and NF-κB in the diabetic rats compared to the diabetic-treated group and the control group. However, the elevated levels of these parameters reversed on co-administration of DIA compared to diabetic-untreated rats (Table 4).

**Table 4:** Effect of DIA on inflammasome, caspase 1, TNFα, and NF-κB

Groups	Inflammasome (pg/mL)	Caspase 1 (pg/mL)	TNFα (pg/mL)	NF-κB (ng/mL)
CON	9.8 ± 1.3	17.4 ± 3.0	29.1 ± 4.0	24.0 ± 4.0
DIA	9.2 ± 1.8	19.3 ± 3.6	26.9 ± 4.9	20.2 ± 3.0
Diabetes	43.9 ± 7.4^ac^	202.5 ± 20.8^ac^	57.5 ± 10.6^ac^	42.2 ± 7.1^ac^
DIA+diabetes	27.4 ± 5.2^ab^	119.9 ± 9.6^ab^	30.6 ± 6.0^b^	35.0 ± 6.3^ab^

Results of current study were for 10 observations represented as mean ± SD. Significance was considered if *p* < 0.05

*CON* control group, *DIA* diacerein, *TNFα* tumor necrosis factor alpha, *NF-κB* nuclear factor kappa B

^a^It was given if significance was found compared to the control group

^b^It was given if significance was found compared to the diabetic group

^c^It was given if significance was found compared to the treated diabetic group.

### *Detection of IL1β using western blotting*

**Fig. 1:**
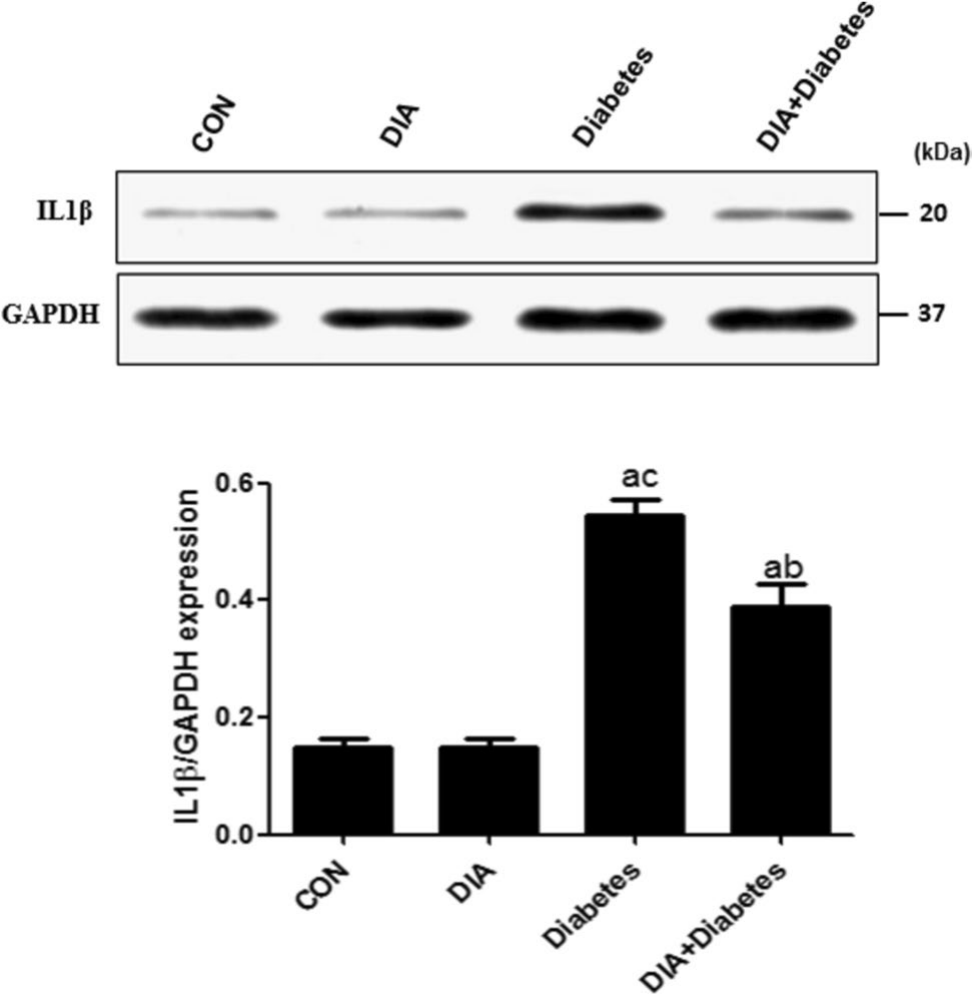
Western blotting of IL1β expression. IL1β expression increased significantly in the diabetic group compared to the control group. However, the diabetic-treated group could significantly decrease IL1β expression if compared to the diabetic-untreated group. Results of the current study were for 10 observations represented as mean ± SD. Significance was considered if *p* < 0.05. ^a^It was given if significance was found compared to the control group. ^b^It was given if significance was found compared to the diabetic group. ^c^It was given if significance was found compared to the treated diabetic group. CON, control group; DIA, diacerein

IL1β level significantly increased in diabetic-untreated rats in comparison with the control group and diabetic-treated rats. However, the DIA co-administered group showed a significant decrease of its level in comparison with the diabetic-untreated group (Fig. 1).

### *Histopathological examination*

**Fig. 2:**
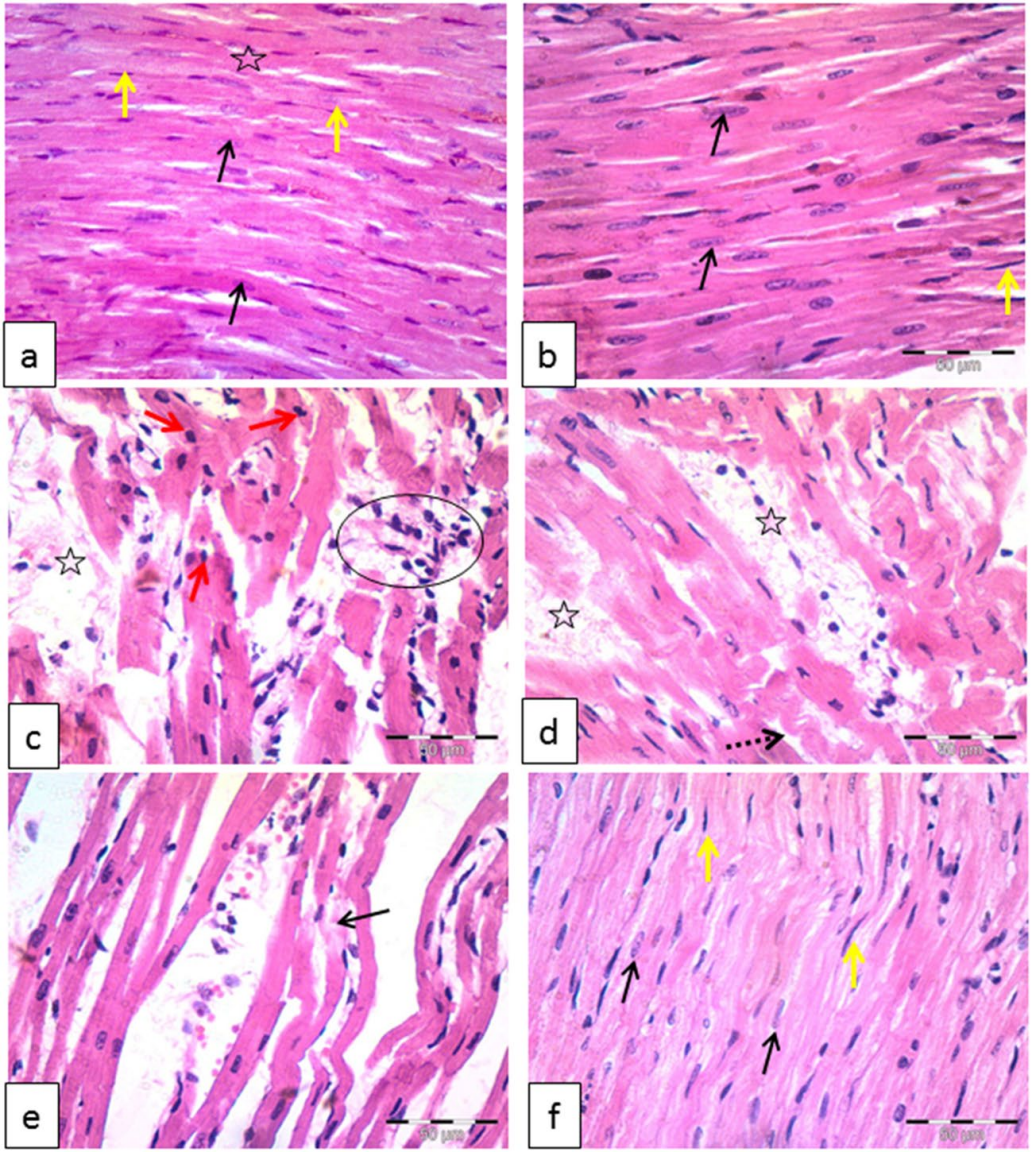
Representative photomicrographs of the control group (**a**) and DIA group (**b**) of left ventricle reveal that individual cardiac myocytes were cylindrical in shape, regularly arranged, branched, and reunite forming a network. Their cytoplasm was acidophilic, crossly striated (star), and had a single central oval and vesicular nucleus (black arrows). Narrow areas of endomysium were observed between cardiac muscle cells. Flat dense nuclei of fibroblasts (yellow arrows) were seen in between of myocytes. Cardiomyocytes in diabetic group (**c**–**e**) showed deformation in sizes and shapes. Areas of fiber loss (star) and disappearance (black arrow) in addition to local inflammatory cellular infiltration were seen (oval). The cytoplasm lost its striations, and some nuclei appeared smaller (red arrows). Additionally, many fibroblast nuclei apparently noticed compared with the control group (oval arrows). Wavy fibers were also noticed (dashed arrow) and extravasated red blood corpuscles (rectangle). In the diabetic-treated group (**f**), cardiomyocytes were apparently improved with normal appearance of the nuclei (black arrows) and scattered fibroblasts (yellow arrows). H&E ×400

Figure 2 shows the histopathological evaluation of the cardiac tissue sections. On examination of the control and DIA groups, individual cardiac muscle cells were cylindrical in shape, regularly arranged, branched, and forming a network. The cytoplasm was acidophilic, striated, and it had a single central oval and vesicular nucleus. Narrow spaces of endomysium were seen between cardiac muscle cells. Flat dense nuclei of fibroblasts were seen in between the myocytes (Fig. 2a, b).

Cardiomyocytes in the diabetic-untreated group (Fig. 2c, d, e) showed deformation in sizes and shapes. Areas of fiber loss and disappearance plus local infiltration with inflammatory cells were observed. The sarcoplasm lost its striations, and some nuclei appeared small and dense. Moreover, many fibroblast nuclei were actually noticed compared with the control group. Wavy fibers were also noticed and extravasated red blood corpuscles.

In the diabetic-treated group (Fig. 2f), cardiac myocytes obviously improved. No foci of muscle loss or inflammatory cell infiltration were observed. Cardiac muscle cells looked normal and branched with narrow intervening intercellular spaces. The cytoplasm was deeply acidophilic and crossly striated. Nuclei appeared normal, and fibroblast cells were apparently much less compared with the diabetic group.

#### Histopathological scoring

**Fig. 3:**
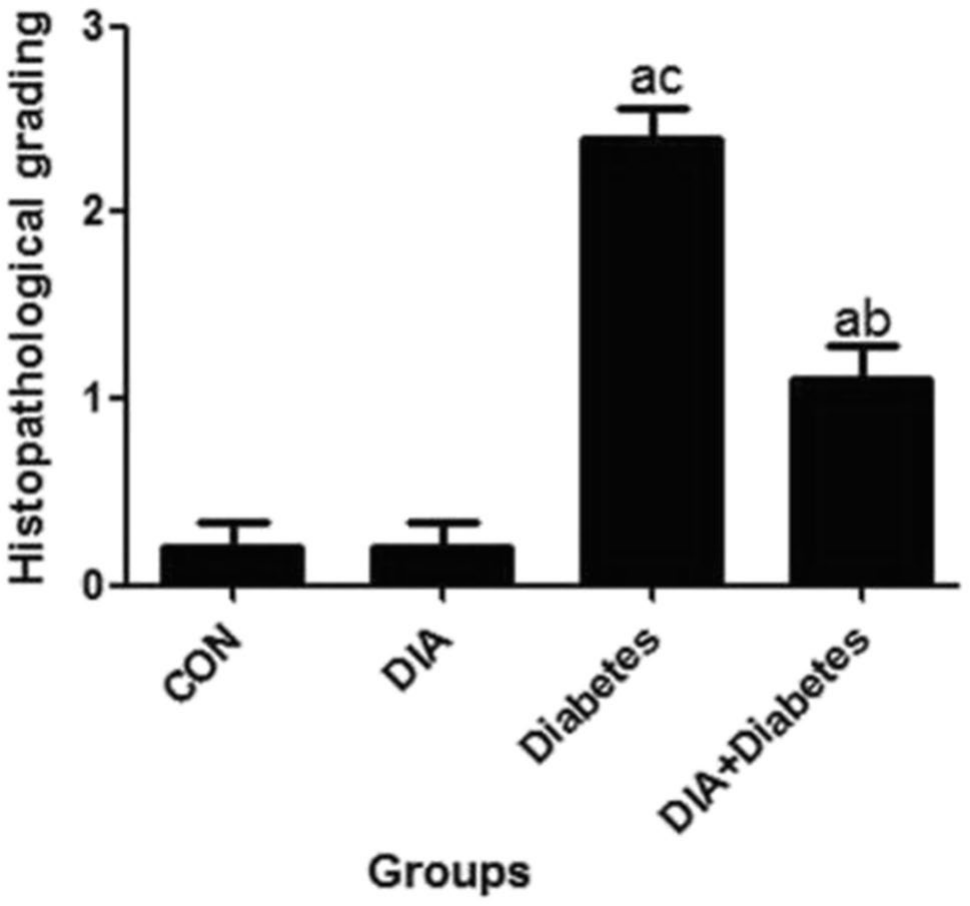
Histopathological scoring. Histopathological grading increased in the diabetic group compared to the control group. However, it decreased in diabetic-treated group if compared to the diabetic-untreated group. Results of the current study were for 10 observations represented as mean ± SD. Significance was considered if *p* < 0.05. ^a^It was given if significance was found compared to the control group. ^b^It was given if significance was found compared to the diabetic group. ^c^It was given if significance was found compared to the treated diabetic group. CON, control group; DIA, diacerein

Results showed significant increase in the scoring of the diabetic-untreated group if compared to the control group. Conversely, the diabetic-treated group revealed significant decrease of scoring if compared to the diabetic-untreated group (Fig. 3).

### *Masson trichrome results*

**Fig. 4:**
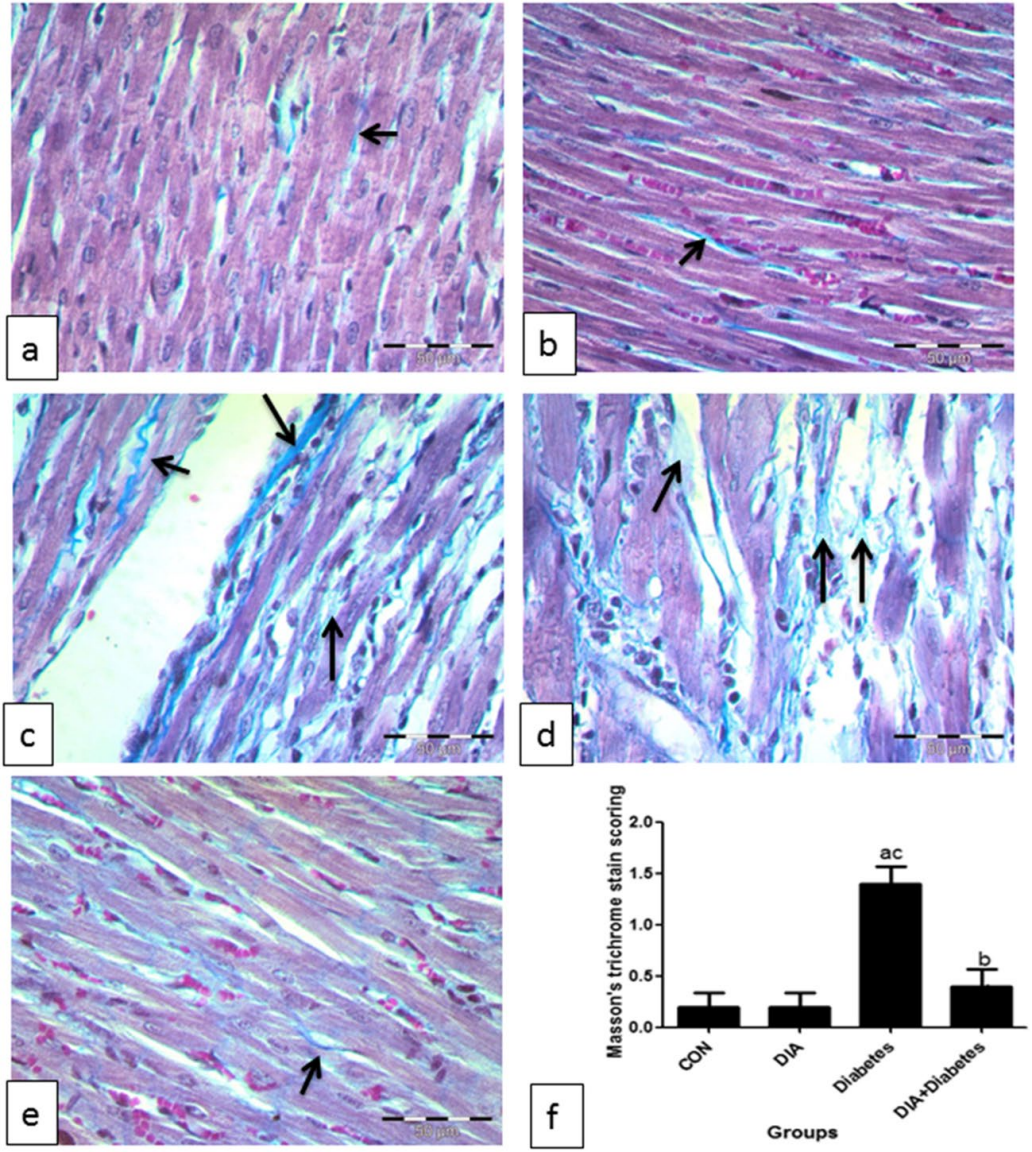
Masson trichrome staining. Representative photomicrographs of the control group (**a**) and DIA group (**b**). Masson trichrome stained sections of the heart showed little collagen fibers among cardiac muscle cells (arrows). The amount of collagen fibers obviously increased among cardiac muscle cells in the diabetic group (**c**, **d**). The amount of collagen fibers clearly decreased in the treated group (**e**) (Masson trichrome ×400). Scoring showed that there was significant increase of Masson trichrome stain in the diabetic group compared to the control group (**f**). However, the diabetic-treated group significantly decreased this scoring compared to the diabetic-untreated group. Results of the current study were for 10 observations represented as mean ± SD. Significance was considered if *p* < 0.05. ^a^It was given if significance was found compared to the control group. ^b^It was given if significance was found compared to the diabetic group. ^c^It was given if significance was found compared to the treated diabetic group. CON, control group; DIA, diacerein

In Fig. 4, the control and DIA groups of Masson trichrome stained sections of the heart showed little collagen fibers in between cardiac muscle cells (Fig. 4a,b). However, the diabetic group sections revealed that the amount of collagen fibers clearly increased in between cardiac muscle cells (Fig. 4c, d). The amount of collagen fibers apparently decreased in the diabetic-treated group (Fig. 4e).

#### Masson trichrome scoring

Figure 4f shows significant increase in scoring of the diabetic-untreated group when compared to the control and diabetic-treated groups. However, the DIA-treated diabetic group showed significant decrease of scoring if compared to the diabetic-untreated group.

### *Immunohistochemical results*

**Fig. 5:**
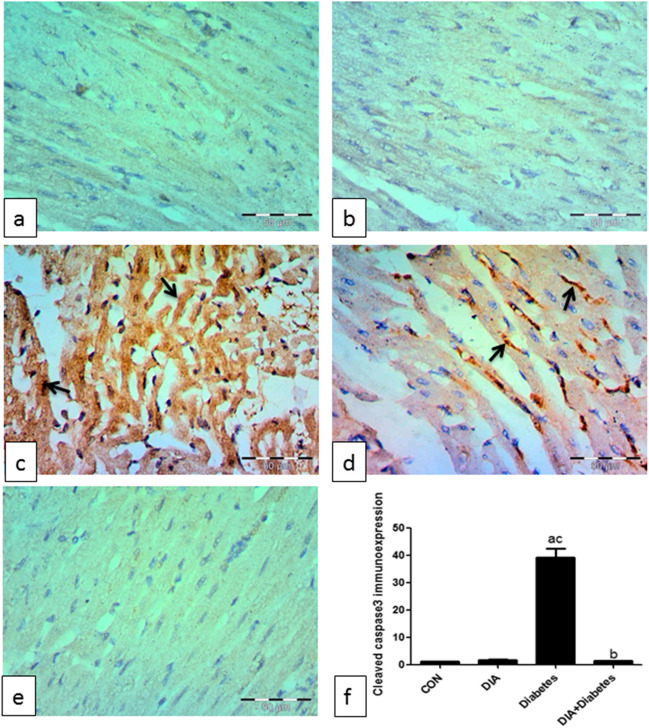
Caspase 3 immunoexpression. Representative photomicrographs of the control group (**a**) and DIA group (**b**) immunohistochemically stained for caspase 3 show negative immunoexpression. The diabetic group shows strong positive immune expression in the cytoplasm and some nuclei (arrows) (**c**, **d**). The treated diabetic group shows noticeable decrease in expression (IHC by caspase 3 antibody ×400) (**e**). Scoring of caspase 3 immunoexpression shows significant increase of caspase 3 immunoexpression in the diabetic group compared to the control group (**f**). However, the diabetic-treated group significantly decreased its expression compared to the diabetic-untreated group. Results of the current study were for 10 observations represented as mean ± SD. Significance was considered if *p* < 0.05. ^a^It was given if significance was found compared to the control group. ^b^It was given if significance was found compared to the diabetic group. ^c^It was given if significance was found compared to the treated diabetic group. CON, control group; DIA, diacerein

Figure 5 shows negative immune expression for anti-caspase 3 antibody in the control and DIA groups (Fig. 5a, b). The diabetic group revealed strong positive immune expression in the cytoplasm and some nuclei (Fig. 5c, d). The diabetic-treated group showed noticeable decrease in expression (Fig. 5e).

#### Caspase 3 immunoexpression scoring

Figure 5f shows significant increase of caspase 3 scoring in the diabetic-untreated group if compared to the control and diabetic-treated group. Though, the diabetic-treated group revealed significant decrease of scoring if compared to the diabetic-untreated group.

## Discussion

Diabetic cardiomyopathy (DCM) is a serious complication in diabetic patients and still great challenges face the prevention of its hazards. Hence, finding new protective agents to preserve the myocardium is mandatory (Dillmann, 2019). The current study discusses the possible beneficial role of DIA to avert DCM in type I diabetes and explores the responsible mechanisms in mediating its effect.

DCM was confirmed in our study as the diabetic group had increases in FBG, HbA1c, and cardiac enzymes with typical features of cardiomyopathy in the histopathological examination. Moreover, there were significant increases in heart weights, BP, inflammasome, Ang II, caspase 1, IL1β, TNFα, NF-κB, caspase 3, and MDA, but significant decreases in GSH and TAC.

Oxidative stress triggered by hyperglycemia is the cornerstone in mediating such illness with dis-balance of the oxidant/antioxidant state inside the cell accompanied with lipid peroxidation of cardiac cell membrane, DNA damage, and finally cell death (Dillmann, 2019, Lorenzo-Almorós et al., 2022). The first line of cell defense against the excessively released free radicals is the intracellular antioxidant enzymes that protect the cell components from the harmful oxidative damaging effect. In addition, GSH is required for initiation and maintenance of different cell processes and regulation of the thiol-redox status, it also detoxifies the released ROS explaining its diminished level in cardiac tissue (Oestreicher and Morgan, 2019). The most dependable indicator of membrane lipid peroxidation is MDA. Its tissue level increased in cardiac tissue but GSH and TAC decreased reflecting the occurrence of oxidative stress damage. These results are in accordance with previous studies (Dillmann, 2019, Oestreicher and Morgan, 2019, Lorenzo-Almorós et al., 2022).

Destruction of cell membrane due to the injurious effect of free radicals causes release of the intracellular cardiac enzymes to the blood stream including CK-MB, LDH, and troponin I; the latter is one of the most essential contractile molecules in the cardiac apparatus that could activate the process of actin over myosin sliding. It is a key diagnostic biomarker of cardiac injury. Besides that, CK-MB is highly sensitive to the occurrence of any form of heart injury (Bugger and Abel, 2014, Chueakula et al., 2018, Wang et al., 2021, Wang et al., 2022a). Our results are in accordance with these data as we found significant increase of the cardiac enzymes in the diabetes type I-induced group due to the associated cardiac injury and release of the intracellular cardiac enzymes.

There are other molecular and cellular mechanisms accounting for DCM. Hyperglycemia and hyperlipidemia associated with changes in insulin level lead to oxidative stress which in turns induce a sort of chronic inflammation that can lead to mitochondrial dysfunction, endoplasmic reticulum stress, and endothelial dysfunction (Singh, 2014). Moreover, hyperglycemia in diabetes can cause disturbances of the immune system, and it is known that the innate immune system is an initial barrier that protects the cell from different stressors and restores tissue homeostasis; however, its over activation can predispose to metabolic diseases *(*Berbudi et al., 2020*)*.

The NLRP3 inflammasome is a critical part of the innate immune system that initiates and propagates inflammatory responses in diabetes (Ding et al., 2022). It was identified that NLRP3 inflammasome is critically involved in the pathogenesis and progression of both type 1 and type 2 diabetes, as its activation leads to stimulation of NF-κB, TNF α, IL1β, and IL-18. This signaling cascade is actively involved in the pathogenesis of diabetes and its complications especially DCM (El Hayek et al., 2021). As a final destination, this cascade leads to a disturbed balance of apoptotic and anti-apoptotic factors, DNA damage, and cell death (Duewell et al., 2010, Huang et al., 2016, Hu et al., 2021).

Upregulation of the apoptotic cascade is a sequence of oxidative stress and inflammation that leads to imbalance of anti-apoptotic and pro-apoptotic factors. It is evaluated mainly by measuring caspase 3 which is the most reliable indicator of apoptosis including both extrinsic and intrinsic pathways (Dhalla et al., 2020, He et al., 2021, Jangsiripornpakorn et al., 2022, Wang et al., 2022b). This is approved in our model that showed significant increases of inflammasome, caspase 1, IL1 β, FBG, HbA1c, and heart weight with histopathological changes in the form of inflammation, edema, inflammatory cell infiltration, and cardiomyopathic changes causing increased heart weight in diabetic rats. Moreover, there was a strong positive expression of caspase 3 indicating increased apoptosis in cardiomyocyte that is greatly involved in the process of compensated to decompensated ventricular dysfunction of diabetic heart, as dead cardiomyocytes are exchanged by extracellular matrix components leading to collagen deposition and myocardial fibrosis (Chueakula et al., 2018, Dewanjee et al., 2021, Nakamura et al., 2022).

Cardiac fibrosis is one of the unique fundamental features of DCM. The elevated NLRP3 inflammasome could exacerbate cardiac fibrosis and endorse hyperglycemia-induced fibrosis (Ding et al., 2022); this is the case in our study as the cardiac tissue of diabetic rats had histological alterations in the form of cellular death, disorganization of cardiac myofibrils, mononuclear inflammatory cell infiltration, and nuclear shrinkage which are features of DCM as detected in previous studies (Cosyns et al., 2007, Thent et al., 2012). Collagen deposition was noticed in Masson’s trichrome stained sections of the diabetic group compared to the control group representing the presence of interstitial fibrosis which can disturb the physiological cardiac functions (Heidarizadi et al., 2022).

It was documented that diabetes is accompanied with overactivity of the sympathetic nervous system, release of catecholamines, upregulation of renin angiotensin system, and higher level of Ang II and aldosterone; the main factor responsible for hypertrophy and remodeling of the myocardium. Thus, the increased level of these vasoactive hormones due to insulin deficiency produces marked defect in the metabolic processes and cardiac remodeling which leads to enhancement of oxidative stress (Vaseghi and Shivkumar, 2008, Paolillo et al., 2013, Dhalla et al., 2020, Tan et al., 2020). This is supported with our findings as we found significant increases of Ang II level with increase in the heart weights in the diabetic group due to hypertrophy and remodeling of the cardiac muscle.

DIA could inhibit the process of synthesis and activity of various pro-inflammatory cytokines such as IL1β, TNF-α, NF-κB, and IL6 and therefore suppress inflammation, apoptosis, and fibrosis with its antioxidant effect causing elevation of the cardiac GSH and TAC associated with marked reduction of MDA content. These findings are compatible with several studies which reported that DIA counteracts ROS, and it has a powerful antioxidant capability in various body organs (El-Sherbiny et al., 2021, Ding et al., 2022, Mohamed Kamel et al., 2022). Prevention of inflammation and oxidative damage protects the cell wall and prevents leakage of the cardiac enzymes as there were significant decreases of cardiac enzymes in serum with normalization of the histopathological features. Moreover, different studies approved that DIA is beneficial for glycemic control, and it can be used as a complementary therapy for diabetes. C-reactive protein, HbA1c levels, and insulin secretion significantly improved during administration of DIA (Jia et al., 2018, Abdel-Aziz et al., 2021, El-Sherbiny et al., 2021, Martorell et al., 2021, Refaie et al., 2022, Silva et al., 2022, Wang et al., 2022b, Xu et al., 2022). Thus, controlling different pathways of DCM upon using DIA leads to decrease in FBG, HgA1c, and downregulate renin angiotensin system and Ang II level.

These pharmacological and physiological properties of DIA explain the observed protective effect and improvement of diabetic cardiomyopathy in juvenile rats. The cardioprotective effect of DIA was approved previously in different models that support our findings as it was able to ameliorate Ang II-induced cardiomyopathy, stress-associated cardiac changes, and different forms of anti-cancer drugs induced heart damage. These beneficial effects of DIA could be attributed to its anti-inflammatory, antioxidant, and anti-apoptotic properties along with its ability to modulate different pro-inflammatory cytokines especially IL1β and improve various forms of metabolic disturbances and cellular changes (Jia et al., 2018, Abdel-Aziz et al., 2021, Agarwal et al., 2021, Silva et al., 2022, Xu et al., 2022).

## Conclusion

Diacerein (50 mg/kg/day) could prevent diabetic cardiomyopathy-induced injury in juvenile rats mostly due to modulation of inflammasome/caspase 1/interleukin 1β pathway, besides its antioxidant, anti-apoptotic, and anti-inflammatory properties.

Our study is the first step in exploring the probable protective role of DIA in diabetic cardiomyopathy, and it may pave the way to its clinical benefit for those cases. However, more researches are recommended to evaluate its role in diabetic patients, and a long-term follow-up should be considered as well.

## Author Contribution

MMMR, SS, EAA-H, and ZHM: conceptualization, methodology, visualization, investigation, writing—original draft preparation, reviewing and editing. HHM and AMAB: methodology, data curation, analysis, writing—original draft preparation, reviewing and editing. The authors declare that all data were generated in-house and that no paper mill was used.

### Supplementary information

ESM 1 (DOCX 61 kb)

ESM 2 (PNG 222 kb)

High resolution image (TIF 46 kb)

## Data Availability

No datasets were generated or analysed during the current study.

## Abbreviations

Ang II: Angiotensin II
BP: Blood pressure
CK-MB: Creatine kinase-MB
DCM: Diabetic cardiomyopathy
DIA: Diacerein
FBG: Fasting blood glucose level
GSH: Reduced glutathione
HbA1c: Glycosylated hemoglobin
IL1β: Interleukin 1β
IL6: Interleukin 6
LDH: Lactate dehydrogenase
MDA: Malondialdehyde
NF-κB: Nuclear factor kappa-B
NLRP3: NLR family pyrin domain containing 3
NLRs: Nod-like receptors
ROS: Reactive oxygen species
STZ: Streptozotocin
TAC: Total antioxidant capacity
TNFα: Tumor necrosis factor-α
