# Supplementary material for: Cardioprotective role of diacerein in diabetic cardiomyopathy via modulation of inflammasome/caspase1/interleukin1β pathway in juvenile rats
Source: Naunyn Schmiedebergs Arch Pharmacol. 2024 Jan 15;397(7):5079–91. doi: 10.1007/s00210-023-02921-8 (PMC11166746; doi:10.1007/s00210-023-02921-8)

"**Molecular and cellular protective role of diacerein in diabetic cardiomyopathy via immune modulation of inflammasome/caspase1/interleukin1β pathway in juvenile rats**"

**Marwa M. M. Refaie^1^, Hanaa Hassanein Mohammed^2^, Elshymaa A. Abdel-Hakeem^3*^, Asmaa M.A. Bayoumi^4^**, **Zamzam Hassan Mohamed^5^, Sayed Shehata^6^**

"Journal of *Naunyn-Schmiedeberg's Archives of Pharmacology*

**Uncropped full Western blot of IL-1b**


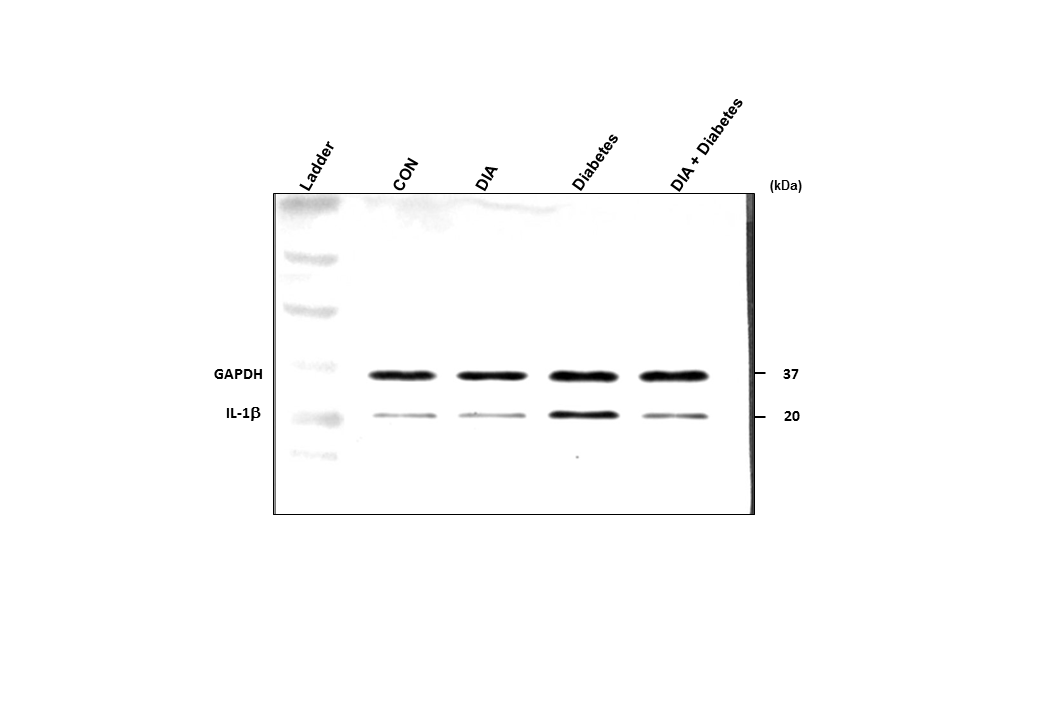

Supplement: Supplementary file 1 — (DOCX 61 kb) [file 210_2023_2921_MOESM1_ESM.docx]
