# Supplementary figures and images for: Cardioprotective role of diacerein in diabetic cardiomyopathy via modulation of inflammasome/caspase1/interleukin1β pathway in juvenile rats
Source: Naunyn Schmiedebergs Arch Pharmacol. 2024 Jan 15;397(7):5079–91. doi: 10.1007/s00210-023-02921-8 (PMC11166746; doi:10.1007/s00210-023-02921-8)

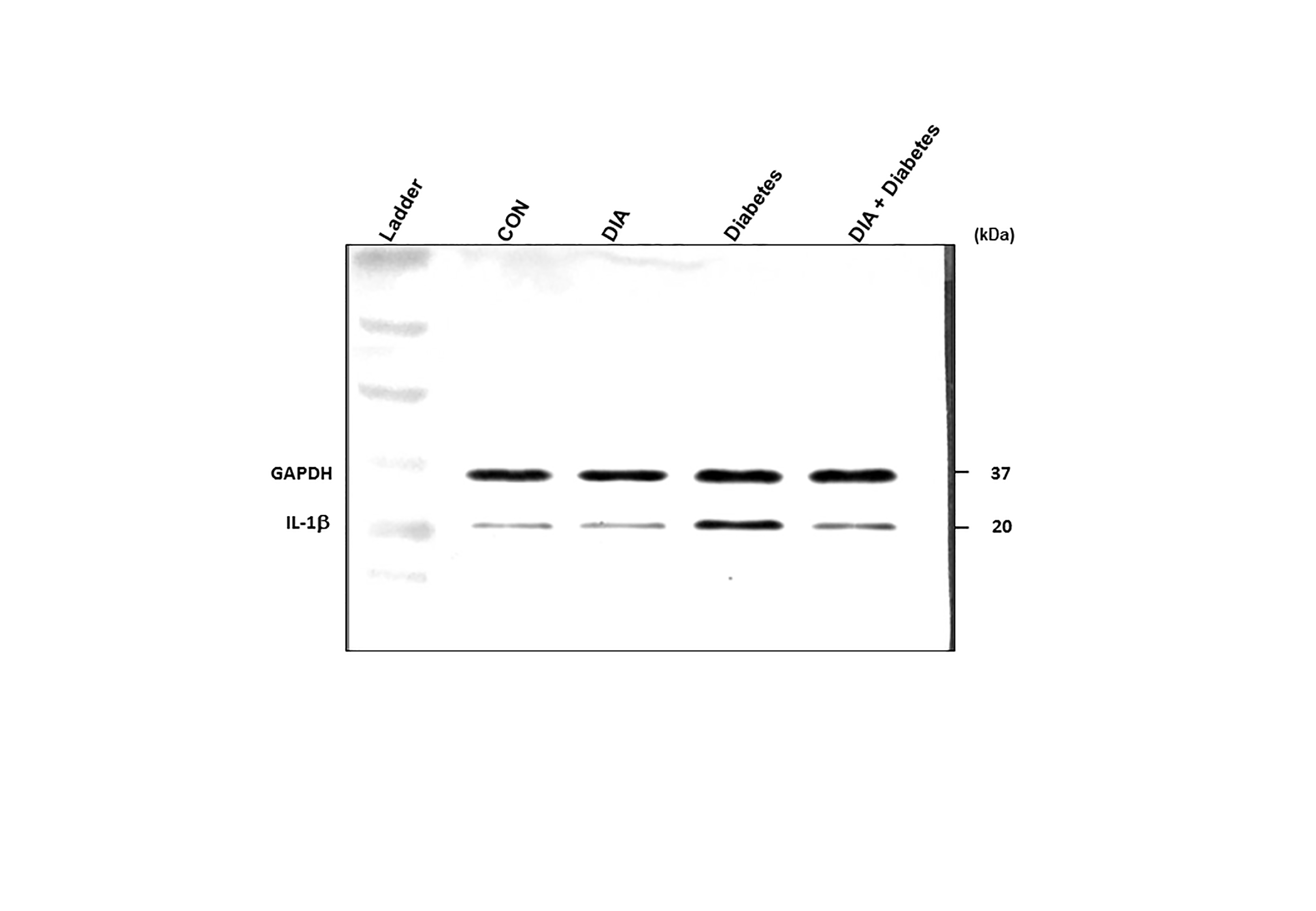

Supplement: Supplementary file 2 — (PNG 222 kb) [file 210_2023_2921_Fig6_ESM.png]

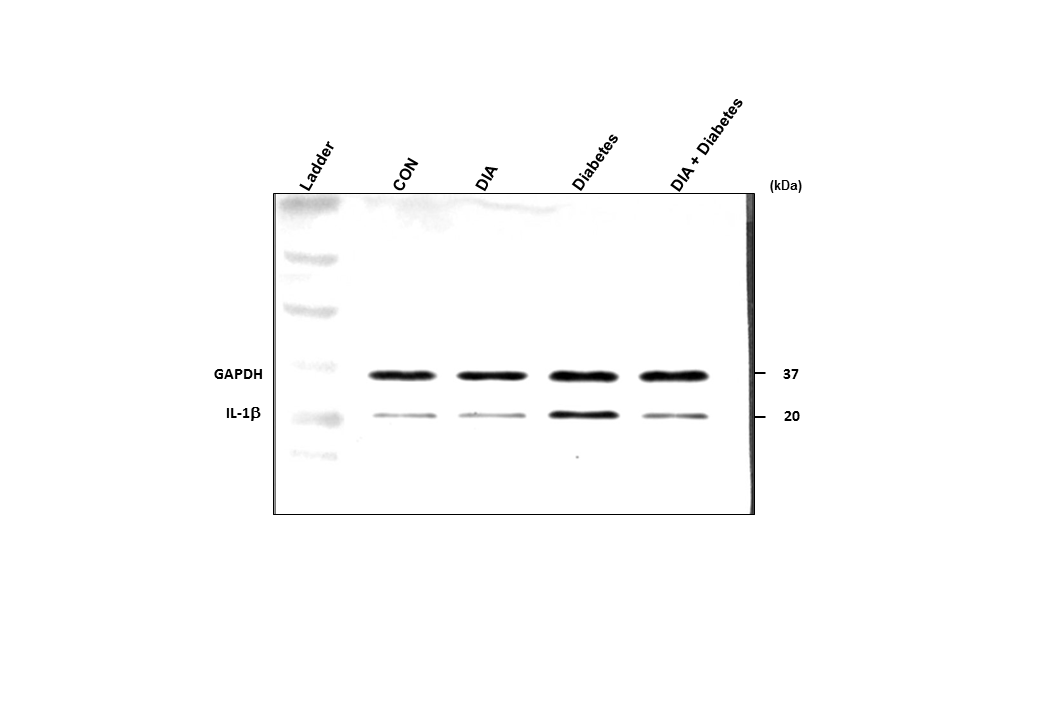

Supplement: Supplementary file 3 — High resolution image (TIF 46 kb) [file 210_2023_2921_MOESM2_ESM.tif]
